# A Series of Rare-Earth Metal-Based Coordination Polymers: Fluorescence and Sensing Studies

**DOI:** 10.3390/s24216867

**Published:** 2024-10-25

**Authors:** Nian-Hao Wang, Jin-Mei Liu, Bin Tan, Zhao-Feng Wu

**Affiliations:** 1 College of Chemistry, Fuzhou University, Fuzhou 350116, China; wangnianhao@fjirsm.ac.cn (N.-H.W.); liujinmei@fjirsm.ac.cn (J.-M.L.); 2 State Key Laboratory of Structural Chemistry, Fujian Institute of Research on the Structure of Matter, The Chinese Academy of Sciences, Fuzhou 350002, China; 3 Fujian College, University of Chinese Academy of Sciences, Fuzhou 350002, China

**Keywords:** FL-CPs, rare earth metals, dual emission, ratiometric sensing

## Abstract

Ratiometric fluorescent sensing based on dual-emitting fluorescent coordination polymers (FL-CPs) has attracted intense attention due to their sensing accuracy and easy visualization when compared with sensing relying solely on monochromatic FL-CPs. In this work, a series of rare-earth metal-based CPs, formuled as [(CH_3_)_2_NH_2_][Ln(bpdc)_2_] (Ln^3+^ = Y^3+^, Eu^3+^ and Tb^3+^, H_2_bpdc = biphenyl-4,4′-dicarboxylic acid), are presented, which show dual emission aroused from the Ln^3+^ ions and the inefficient intermolecular energy transfer from ligands to Ln^3+^ metals. For clarity, the as-made Ln-CPs are named Eu-bpdc, Tb-bpdc, and Y-bpdc based on the corresponding Ln^3+^. Notably, Eu-bpdc, presented as an example, could be used as FL sensing material ratiometric to Fe^3+^ ions. The ratio of FL intensity of Eu^3+^ ions to bpdc^2−^ ligands (*I*_415_/*I*_615_) showed a good linear relationship with the concentrations of Fe^3+^ ions. Moreover, the detection process could be visibly monitored through a change from purple to blue when Eu-bpdc was used as an FL proble. This work provides a good example for exploring visibly ratiometric sensors based on FL-CPs.

## 1. Introduction

Metal ions play important roles in biological metabolism. Regularly, metal species usually maintain a trace concentration level in biological systems, but an excess or deficiency in specific metals usually brings great harm to the biological environment and even threatens life [1]. Fe^3+^, as one essential element, plays a great part in living organisms. A lack of Fe^3+^ can cause hemochromatosis, while an excess in Fe^3+^ can induce Alzheimer’s disease [2,3,4]. Therefore, many methods have been developed to monitor the concentration level of Fe^3+^ ions. Thus far, HPLC, ion-selective electrodes, ion chromatography, etc., are the conventional ways of determining Fe^3+^ [5]. Nevertheless, professional technicians, expensive instruments, and relatively time-consuming data analyses are inevitably needed, inconveniencing the use of testing experiments especially in undeveloped areas. Therefore, convenient, rapid-response, and highly selective and sensitive detection ways for determining Fe^3+^ ions are desirable.

Optical sensors, particularly fluorescence (FL) sensors, are particularly attractive for sensing due to their simple operation, low cost, and good sensitivity [6]. The past two decades have witnessed great progress made in FL coordination polymers (FL-CPs) and metal–organic frameworks (FL-MOFs), especially those assembled from rare earth metals due to their bright, narrow characteristic FL emission band and long emission lifetime [7,8,9,10,11]. Multiple FL mechanisms have been raised for the designation of FL-CPs, such as metal and ligand cantered emission, energy transfer between ligands and metal ions, and guest-induced emission [12,13,14]. As a result, FL sensors based on CPs can be custom-made due to their designable characteristics, which have already been well developed for the sensing of toxic cations, anions, molecules, and so forth [15,16,17]. Thus far, FL-CP sensors are regularly dependent on monitoring the FL signal of the monochromatic emission, and their sensing sensitivity and accuracy might be interfered with by external factors, e.g., excitation and emission intensity, probe and analyte concentration, and temperature, to name a few [18,19]. Therefore, the designation of FL-CPs with ratiometric sensing is proposed to alleviate such problems [20,21].

In the ratiometric sensing process, FL-CPs exhibit dual-emitting FL under one excitation, in which one single emission will be used as the inner reference, and the linear relationship between the ratio of the dual-emission intensity and the concentration of the analyte is established to keep the probing accuracy. Moreover, changing the FL signals of the dual-emitting centers could tune the emission color of the CPs, causing the sensing process to be conveniently monitored by the naked eye [22,23,24]. For example, Chen. B. and Qian. G. et al. presented a porous lanthanide MOF ZJU-88 that exhibited the strong characteristic transitions of Eu^3+^ at 594, 615, and 699 nm. A perylene dye molecule could be loaded into ZJU-88 to form a ZJU-88⊃perylene composite, achieving dual emission with both the characteristic emissions of the perylene dye and Eu^3+^ ions. Along with the increase in the measuring temperature from 20 to 80 °C, the luminescence intensity at 473 nm of the perylene dye in ZJU-88⊃perylene substantially decreased, while the intensity of the characteristic Eu^3+^ emission at 615 nm increased. There was a very good linear relationship between the intensity ratio of *I*_615_/*I*_473_ and the temperature. Therefore, a ratiometric thermometer with high sensitivity was established [22]. Xie. J. et al. prepared an alizarin complexone-modified UiO-66-NH_2_, demonstrating significant changes in color from pale yellow to deep pink, while the fluorescence shifted from light blue to blue violet toward pH values. Based on the visible checking character, this complex was very suitable for detecting the freshness of perishable food [23]. Thus far, ratiometric sensing arrays based on FL-CPs are mostly prepared through post-synthesis manners that tend to suffer from synthesis difficulties [22,23,24,25,26,27]. To date, monomeric FL CPs with dual-emitting characteristics are comparatively rare [28,29].

Herein, three lanthanide FL-CPs [NH_2_(CH_3_)_2_][Ln(bpdc)_2_] (denoted as Ln-bpdc; Ln = Y, Eu, and Tb; H_2_bpdc = biphenyl-4,4′-dicarboxylic acid) are presented. Eu-bpdc and Tb-bpdc exhibit a bright FL with dual-emission bands originating from Ln^3+^ ions and the organic ligand due to the inefficient intermolecular energy transfer from bpdc^2−^ linkers to Ln^3+^ ions. Eu-bpdc exhibits selective ratiometric sensing toward Fe^3+^ ions, by reducing the FL intensity of Eu^3+^ ions while increasing that of the bpdc^2−^ linker. As a result, a ratiometric Eu-bpdc chemical sensor was developed, and a good linear relationship between the ratio of the FL intensity of *I*_415_/*I*_615_ and the concentration of Fe^3+^ ions was established. The sensing process could be easily monitored with the naked eye through color changes from purple to blue. In addition, Eu-bpdc could be stable in water with a wide pH value (2–10), ensuring that the FL sensing can be conducted even in harsh conditions. Many experimental characterizations have been applied to understand the sensing mechanism. This work will provide a good reference for the future designation of a ratiometric sensing matrix based on FL-CPs.

## 2. Materials and Methods

Reagents. All reagents and chemicals were purchased from commercial sources and used without further purification. Ln(NO_3_)_6_·6H_2_O (≥99%, Adamas-beta, Shanghai Titan Chemical Co., Ltd., Shanghai, China); H_2_bpdc (Beijing HWRK Chem Co., Ltd., Beijing, China); N,N-dimetylformamide (DMF, AR, Greagent, Shanghai Titan Chemical Co., Ltd., Shanghai, China).

Syntheses of [(CH_3_)_2_NH_2_][Ln(bpdc)_2_]. A mixture of Ln(NO_3_)_2_·6H_2_O (0.5 mmol, Ln = Y^3+^, Eu^3+^ and Tb^3+^), H_2_bpdc (0.5 mmol), 4.0 mL DMF, 1.0 mL CH_3_OH, and 1.0 mL H_2_O was sealed in a 20 mL vessel, heated at 120 °C for 3 days, and then cooled to room temperature. Crystalline powder of the title Ln-CPs was filtrated, washed with ethanol and CH_2_Cl_2_ several times, and then dried in the air. Crystal samples that were suitable for single-crystal X-ray diffraction analyses were obtained through the method reported in the literature [30].

Physical measurements. Powder X-ray diffraction (PXRD) patterns were recorded on a Rigaku MiniFlex II diffractometer (Rigaku Corporation, Tokyo, Japan) using CuK*α* radiation (λ = 1.54178 Å). A graphite monochromator was used, and the generator power settings were set at 44 kV and 40 mA. Data were collected between 2*θ* and 5–35° with a scanning speed of 1.0°/min. Thermogravimetric (TG) data were collected on a NETZSCH STA449C Analyzer (Netzsch, Bavaria, Germany) with a temperature ramping rate of 10 °C/min from 30 to 800 °C under N_2_ flow. Energy dispersive spectroscopy (EDS) was performed using a JSM-6700F scanning electron microscope (JEOL, Akishima, Japan). UV-vis absorption spectra were measured using a Mapada UV-3100 spectrometer (Shanghai Mapada Instrument Co., Ltd., Shanghai, China). Solid-state FL and FL detections were recorded using a PerkinElmer LS55 FL spectrometer (PerkinElmer Instrument (Shanghai) Co., Ltd., Shanghai, China), and all the FL related experiments were conducted at room temperature.

FL sensing measurements. The as-made crystalline samples of Eu-bpdc were manually ground to obtain fine powders. Then, 2 mg powdered samples were dispersed in 2 mL aqueous solutions of 10^−2^ M metal ions or anions, which were ultra-sonicated to obtain a stable emulsion. The suspension was placed in a quartz cell with a 1 cm width for FL measurements. The FL sensing experiments were conducted by dispersing 2 mg Eu-bpdc in 2 mL water under ultra-sonication, and then 10^−2^ M Fe^3+^ with various concentrations was injected to the resulting suspensions using a pipette. For all the measurements, the dispersed emulsions of compound were excited at 335 nm, while the corresponding emission wavelengths were monitored from 380 to 750 nm. The parameters of the FL spectrometer were kept the same for every sensing experiment.

## 3. Results and Discussions

### 3.1. Crystal Structure Analysis and Basic Measurements

**Figure 1:**
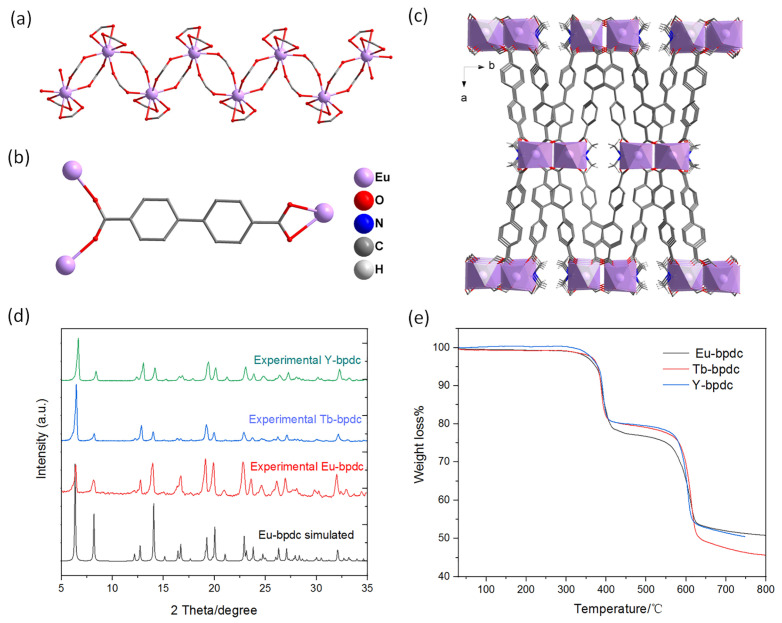
(**a**) The one-dimensional (1D) metal–carboxylic chain with a coordination environment of Eu^3+^ ions in Eu-bpdc. (**b**) The coordination mode of the H_2_bpdc ligand in Eu-bpdc. (**c**) The three-dimensional (3D) structure of Eu-bpdc viewed along the *c* axis. Hydrogen atoms located in the benzene ring are omitted for clarity. (**d**) PXRD patterns of the as-made Eu-bpdc samples. (**e**) TG curves of the as-made Ln-bpdc samples.

The structure of the Eu-bpdc has been reported in a previous study [30], but the Tb- and Y-bpdc are firstly reported in this work. Therefore, Eu-bpdc was selected as an example to provide a brief structure analysis. As depicted in Figure 1a, the Eu^3+^ ion is eight-coordinated by eight carboxylic oxygens from six different COO^−^ groups, in which two are adopting a chelating coordination, while four are assuming a monodentate mode. Each Eu^3+^ is bridged by sharing four carboxylic groups in monodentate coordination to bridge the neighboring Eu^3+^ to form a 1D metal-carboxylic {Eu(COO)_6_}_n_ chain, which is further linked by the bpdc^2−^ ligands to generate a 3D framework, as shown in Figure 1b,c. The dimethylammonium cations are generated from the in situ decomposition of DMF, which are located around the COO^−^ groups to balance the charge of the resultant structure. The purity of the as-made compound was identified by PXRD measurements. Their isostructural characters could be identified by comparing their PXRD patterns, as shown in Figure 1d. As seen in Figure 1e, their structures could be maintained until 300 °C without any weight loss, demonstrating their good thermal stability. The IRs of the as-made compounds are also presented (Figure S1).

### 3.2. Photoluminescence Studies

**Figure 2:**
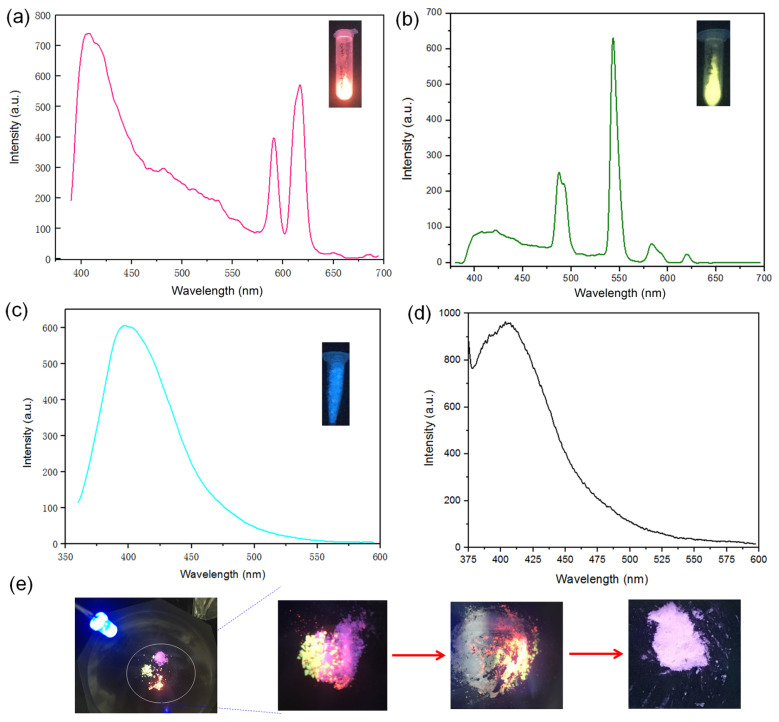
(**a**) Solid-state FL spectra of as-made Eu-bpdc. (**b**) Solid-state FL spectra of as-made Tb-bpdc. (**c**) Solid-state FL spectra of as-made Y-bpdc. Insets are photographs of the corresponding Ln-bpdc samples under 365 nm UV light. (**d**) FL spectra of H_2_bpdc ligand. (**e**) Photographs of the corresponding mixtures of Eu-bpdc, Tb-bpdc, and Y-bpdc under a 365 nm UV lamp. All measurements were conducted at room temperature.

Solid-state FL measurements indicate that the H_2_bpdc ligand exhibits a blue emission maximized at 410 nm under 365 nm excitation. After being assembled into a 3D framework, the resultant Ln-bpdc exhibits a dual emission derived from the characteristic emission of the Ln^3+^ ions (Ln = Eu and Tb) and the bpdc^2−^ linker. As shown in Figure 2a, the main characteristic sharp emission bands for Eu-bpdc around 600 nm are attributed to Eu^3+^, which could be assigned to the ^5^D_0_ → ^7^F*_J_*(*J* = 1–4) transitions: ^5^D_0_ → ^7^F_1_ (around 590 nm), ^5^D_0_ → ^7^F_2_ (around 615 nm), and ^5^D_0_ → ^7^F_4_ (around 685 nm), respectively. While for Tb-bpdc, the four characteristic peaks around 495, 548, 588, and 622 nm can be assigned to the ^5^*D*_4_ → ^7^*F_n_* transitions (*n* = 6, 5, 4, 3, respectively; Figure 2b) [8,9]. The emission maximized around 415 nm could be ascribed to the organic ligand of bpdc^2−^. The ligand-centered emission could be further certified by studying the FL spectra of the isostructural compound Y-bpdc. As seen in Figure 2c, both the FL spectral shape and the emitting band peak maximized around 410 nm for the Y-bpdc are almost the same as that of the bpdc^2−^ ligand (Figure 2d), demonstrating that the dual emission for Eu- and Tb-bpdc are attributed to the FL of the organic ligand and Ln^3+^ ions sensitized by the ligand through the antenna effect but with a lower transfer efficiency [21]. As we know, Tb^3+^ is more difficult to sensitize with the organic ligand than Eu^3+^ ion. So, the bpdc^2−^ in this work represents a good photosensitizer for sensitizing both the Eu^3+^ and Tb^3+^ ions. Therefore, based on an RGB mechanism, the white emission could be generated by mixing Eu-, Tb-, and Y-bpdc with a certain proportion under 365 nm UV light, as shown in Figure 2e.

Interestingly, both the FL of Eu- and Tb-bpdc with dual-emission bands could be monitored by tuning the excitation energy. Taking Eu-bpdc as an example, as depicted in Figure S2a, by increasing of excitation wavelength, the FL intensity of 415 nm increases first under excitation from 310 to 340 nm and then decreases from 340 to 370 nm. Comparatively, the FL intensity of the characteristic of the Eu^3+^ ion shows inconspicuous changes. Therefore, the FL of the Ln-CPs in solid state is dominated by tuning the emission intensity of the bpdc^2−^ ligand. This result further demonstrates that the dual-emitting character of Eu-bpdc is caused by the inefficient energy transfer between the ligand and Eu^3+^ ions in Eu-bpdc. As a result, as shown in the CIE chromaticity diagram, the FL of Eu-bpdc could be monitored from blue to purple with a CIE coordinate of (0.18 0.05) to (0.28 0.18), as shown in Figure S2b. A similar phenomenon is also observed for compound Tb-bpdc. As depicted in Figure S2c, with the increasing excitation wavelength from 320 to 365 nm, the FL intensity originating from the ligand and Tb^3+^ alternatively increases and decreases, resulting in the FL of Tb-bpdc changing from green to blue and then finally emitting green. Reflected in the CIE chromaticity diagram, the CIE coordinate of (0.20 0.30) located in the blue region could gradually tuned to (0.25 0.51) in the green location, as shown in Figure S2d. The excitation-dependent FL of the dual-emitting Eu- and Tb-bpdc caused by the energy transfer between the ligand and Ln^3+^ ions could also be further demonstrated by measuring the FL of Y-bpdc under different excitation wavelengths. As seen in Figure S3, the FL intensity of Y-bpdc decreases by increasing the excitation wavelength from 330 nm to 365 nm, but its emission is only located in the blue region according to its CIE chromaticity diagram. This result demonstrates that the excitation energy dominates the FL of the bpdc^2−^ ligand, which further influences the energy transfer from the ligand to Eu^3+^ or Tb^3+^ ions to tune their FL.

### 3.3. Photoluminescence Sensing Performances

**Figure 3:**
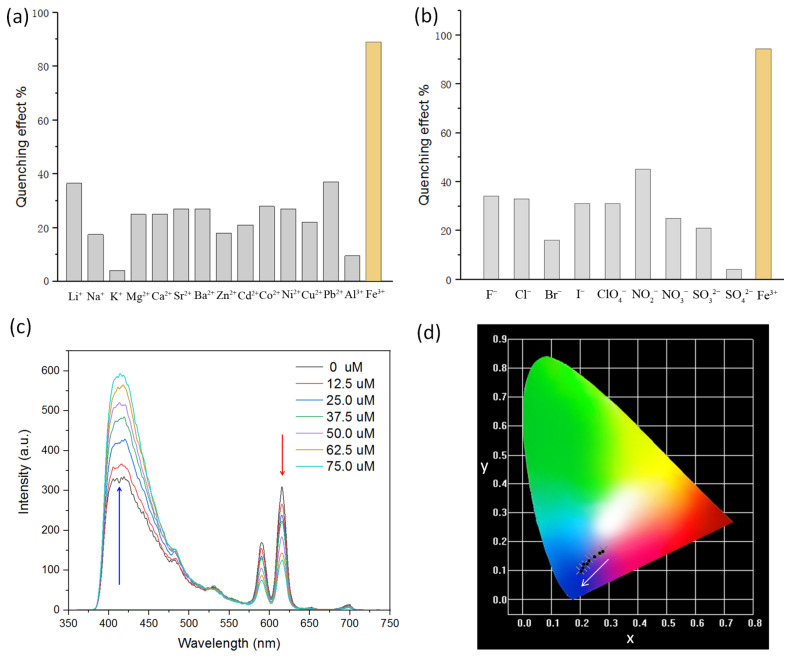
(**a**) FL quenching efficiency of Eu-bpdc toward different metal cations. (**b**) FL quenching efficiency of Eu-bpdc toward varied anions. (**c**) FL spectra of Eu-bpdc with the addition of different concentrations of Fe^3+^. (**d**) CIE chromaticity diagram of Eu-bpdc corresponding to different concentrations of Fe^3+^.

The dual-emitting characters of the as-made Eu-bpdc and Tb-bpdc indicate that they might be potential ratiometric FL sensors in sensing applications. Eu-bpdc as a representative was investigated to present its FL probing performance. The fine powder of Eu-bpdc is dispersed in 10^−2^ M aqueous solutions with different metal ions or anions to form a uniform suspension through sonication treatment. As shown in Figure 3a,b, although Eu-bpdc demonstrates some extent degree of FL intensity changes toward other metal ions, even regularly seen anions, the most significant and obvious FL-enhancing response is observed in the case of Fe^3+^ with high selectivity (Figure S4). By adding Fe^3+^ solution into the FL emulsion, the characteristic emission of the Eu^3+^ ion shows an obvious decrease in extent, while the FL intensity of the bpdc^2−^ linker increases more rapidly as a function of increasing Fe^3+^ concentrations, as shown in Figure 3c. The FL signal of *I*_615_ is quenched by more than 50% with only an addition of 50.0 μM Fe^3+^, demonstrating its good sensing sensitivity (Figure 3c). By alternatively increasing and reducing the FL emission intensity of the Eu^3+^ and bpdc^2−^ ligand, the emitting color could be monitored from purple to blue with the addition of Fe^3+^ ions. From the CIE chromaticity diagram for Eu-bpdc emulsion, the sensing process could be visibly checked by changing the CIE coordination from (0.28 0.17) to (0.20 0.10), as shown in Figure 3d. Therefore, using the emission of Eu^3+^ as an inner reference, the FL intensity ratio of *I*_415_/*I*_615_ shows a good liner relationship with the concentration of the Fe^3+^ ions (R^2^ = 0.96886), and then an Eu-bpdc based ratiometric FL sensor for Fe^3+^ was developed (Figure S5a). Stern–Volmer (SV) analysis calculated from the SV equation (*I*_0_/*I* = 1 + *K*_sv_[M]) was applied to check the sensitivity of the ratiometric FL array [31,32]. In this work, the FL intensity of *I*_615_ was used to detect the quenching effect for Fe^3+^, and the *K*_sv_ value was calculated to be 1.87 × 10^4^ M^−^, demonstrating the high sensing efficiency (Figure S5b). Accordingly, the detection limit (LOD) was calculated to be 10.5 μM, obtained from the ratio of 3*δ*/slope, in which *δ* is the standard deviation of the FL intensity of the blank solution [33,34]. The LOD of Fe^3+^ reported here is comparable to that of other reported rare earth metals, transition metals, and even 3d-4f mixed metal-based CP sensors that can be used to conduct sensing experiments in H_2_O (Table 1).

**Figure 4:**
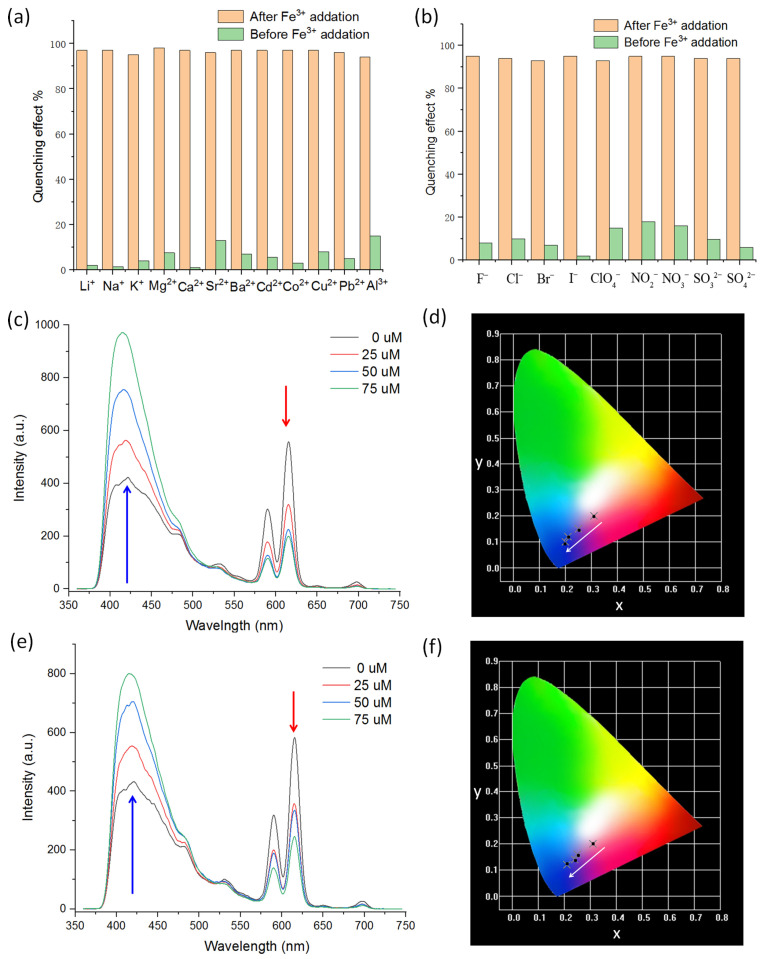
(**a**) The FL quenching efficiency of Eu-bpdc upon an addition of Fe^3+^ in the absence and presence of different metal cations. (**b**) The FL quenching efficiency of Eu-bpdc upon an addition of Fe^3+^ in the absence and presence of varied anions. (**c**) The FL spectra Eu-bpdc dispersed in 10^−2^ M Ca^2+^ solution with an addition of Fe^3+^ ions. (**d**) The corresponding CIE chromaticity diagram of Eu-bpdc in 10^−2^ M Ca^2+^ solution with an addition of Fe^3+^ ions. (**e**) The FL spectra Eu-bpdc dispersed in 10^−2^ M Al^3+^ solution with an addition of Fe^3+^ ions. (**f**) The corresponding CIE chromaticity diagram of Eu-bpdc in 10^−2^ M Al^3+^ solution with an addition of Fe^3+^ ions.

**Figure 5:**
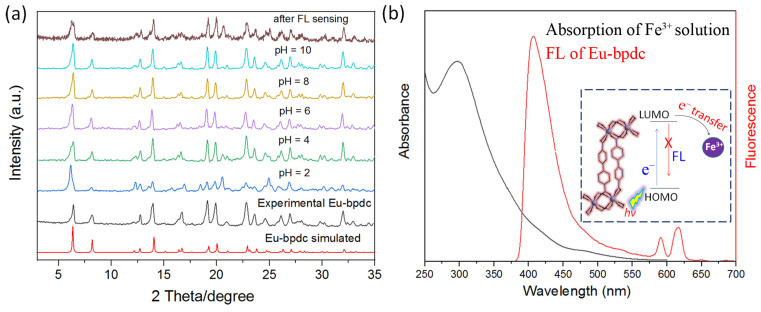
(**a**) The PXRD of the Eu-bpdc immersed in water with different pH conditions and after Fe^3+^ sensing. (**b**) The absorption spectra of the Fe^3+^ solution and the FL spectra of the as-made Eu-bpdc. The inset is the FL quenching diagram of using Eu-bpdc as an FL sensing material for Fe^3+^ ion.

The anti-interference ability is also an important parameter for an FL sensor. Figure 4a,b indicate that the presence of other competing analytes has insignificant effects on the selectivity of FL sensing of Eu-bpdc toward Fe^3+^, using *I*_615_ as a reference (Figures S6 and S7). As we know, cations (e.g., Ca^2+^ and Al^3+^) are regularly seen in natural water systems. Therefore, the ratiometric FL sensing ability of Eu-bpdc to Fe^3+^ within these competing analytes is further investigated. Notably, Eu-bpdc maintains a good ratiometric FL sensing performance toward Fe^3+^, regardless of dispersion in divalent Ca^2+^ or trivalent Al^3+^ solutions (Figure 4c–e). From their chromaticity diagram, the sensing progress could still be visible from purple to blue (Figure 4d,e). This result indicates the good anti-interference capacity of Eu-bpdc, suggesting its potential sensing application to Fe^3+^ in natural water environments. Water tolerant stability is also an important characteristic for an FL sensor as the sensing operation might be conducted in water with harsh conditions. Then, the Eu-bpdc was further immersed in water with varied pH values ranging from 2 to 10 to check its stability. As depicted in Figure 5a, the PXRD of the water treated samples are almost the same as those of the as-made sample, demonstrating the excellent water tolerance of Eu-bpdc even in harsh pH conditions. The Y- and Tb-bpdc also show good tolerance to water in varied pH values (Figures S8 and S9). The sample after FL sensing was also collected to check the structural stability. The PXRD of the collected sample were the same as those of the as-made sample, indicating that the FL quenching is not caused from the structure collapse (Figure 5a). EDS mapping indicates that there are no residual Fe^3+^ in the collected Eu-bpdc sample after FL sensing, demonstrating that the FL quenching is not increased from the reaction between Fe^3+^ and Eu-bpdc (Figure S10). The paramagnetic high-spin Fe^3+^ ion with a d^5^ configuration has a strong electron-withdrawing ability. According to previous studies, the main mechanism of detection for Fe^3+^ as the FL quencher might be that upon excitation by photons, the excited electrons of Eu-bpdc are transferred to Fe^3+^ ions to quench its FL (inset of Figure 5b) [39,47]. As depicted in Figure 5b, the maximum absorption of aqueous Fe^3+^ used as a quencher here is located around 320 nm with an extension to the visible region, exhibiting some extent overlap with the 410 nm of the organic ligand and the 415 nm emission region of Eu-bpdc, indicating the energy transfer between the bpdc^2−^ in Eu-bpdc and Fe^3+^, which would also facilitate the quenching of its luminescence [48,49,50].

## 4. Conclusions

In conclusion, a series of dual-emitting Ln-bpdc CPs (Ln = Eu, Tb and Y) were presented, in which the Eu-bpdc was presented as an example of a visibly ratiometric FL sensor for probing Fe^3+^ ions. The compound exhibited excellent selective and sensitive sensing performances for Fe^3+^ ions operating in water. The good linear relationship between its FL intensity ratio of *I*_415_/*I*_615_ and the concentrations of Fe^3+^ ions was established (R^2^ = 0.96886), ensuring the sensing accuracy of this ratiometric sensing matrix. The LOD of the Eu-bpdc toward Fe^3+^ ions was below those of other sensing materials based on FL Ln-CPs. Moreover, the Eu-bpdc exhibited a good tolerance to water with a wide pH range, suggesting its potential sensing application in harsh conditions. Various experimental characterizations were conducted to understand the sensing mechanism. In the future, we will focus on the designation of more ratiometric FL sensing materials based on CPs.

## Figures and Tables

**Table 1:** Selected FL CP sensors for Fe^3+^ operating in water for comparison study.

Compound Names	*K*_sv_ Value/M^−^	LOD/μM	References
Eu-bpdc	1.87 × 10^4^	10.5	This work
EuL_3_	4.1 × 10^3^	--	[35]
Eu^3+^@MIL-53-COOH (Al)	5.12 × 10^3^	0.5	[36]
Tb-DSOA	3.54 × 10^3^	--	[37]
[Eu(HL)(H_2_O)_3_]·H_2_O	0.88 × 10^4^	2.0	[38]
[Cd(5-asba)(bimb)]	1.78 × 10^4^	--	[39]
[Eu_2_(pdba)_3_(H_2_O)_3_]·2H_2_O	6.53 × 10^4^	--	[40]
[Eu_3_(pdba)_4_(H_2_O)_4_]·5H_2_O	5.76 × 10^4^	--	
[Zn(L)]·2.7DMF	4.5 × 10^3^	1.66	[41]
[Cd(ATA)(L)]·2H_2_O	3.84 × 10^3^	3.76	[42]
[Zn(ATA)(L)]·2H_2_O	0.56 × 10^3^	1.77	
[Zn_2_(L)(5-AIP)_2_]·3H_2_O	4.475 × 10^3^	1.29	[43]
Zn-DTA	8.4 × 10^3^	0.82	[44]
Cd-DTA	6.42 × 10^3^	1.07	
[Zn(OBA)_2_(L1)·2DMA	4.22 × 10^4^	1.06	[45]
[Ca(H_2_O)_6_][Cu_6_Ca_2_(mna)_6_(H_2_O)_6_]·H_2_O	1.38 × 10^5^	0.32	[46]

Note: -- means not mentioned.

## Data Availability

Data are contained within the article and Supplementary Materials.

## Supplementary Materials

The following supporting information can be downloaded at: https://www.mdpi.com/article/10.3390/s24216867/s1, Figure S1: The FT-IR spectra for the as-made Ln-bpdc; Figure S2: (a) The excitation wavelength dependent FL spectra of the as-made Eu-bpdc measured at room temperature. (b) The CIE chromaticity diagram of compound Eu-bpdc corresponding to different excitation wavelength. (c) The excitation wavelength dependent FL spectra of the as-made Tb-bpdc measured at room temperature. (b) The CIE chromaticity diagram of compound Tb-bpdc corresponding to different excitation wavelength; Figure S3: (a) The excitation wavelength dependent FL spectra of the as-made Y-bpdc. (b) The CIE chromaticity diagram of Y-bpdc corresponding to different excitation wavelength; Figure S4: (a) The FL spectra of Eu-bpdc dispersed in varied metal ion solutions. (b) The FL spectra of Eu-bpdc dispersed in varied anion solutions; Figure S5: (a) The linear relationship between the FL intensity ratio (*I*_415_/*I*_615_) and the concentrations of Fe^3+^. (b) The *K*_sv_ curve of Eu-bpdc towards Fe^3+^ ions by using *I*_615_ as a reference. The data are collected at least two times to keep accuracy; Figure S6: The FL spectra of Eu-bpdc upon addition of Fe^3+^ solution in the absence and presence of different metal cations; Figure S7: The FL spectra of Eu-bpdc upon addition of Fe^3+^ solution in the absence and presence of different anions; Figure S8: The PXRD of the Y-bpdc immersed in water with different pH conditions; Figure S9: The PXRD of the Tb-bpdc immersed in water with different pH conditions; Figure S10: The EDS mapping of Eu-bpdc after Fe^3+^ sensing.
