# Supplementary material for: A Series of Rare-Earth Metal-Based Coordination Polymers: Fluorescence and Sensing Studies
Source: Sensors (Basel). 2024 Oct 25;24(21):6867. doi: 10.3390/s24216867 (PMC11548555; doi:10.3390/s24216867)
Supplement: Supplementary file 1 [file sensors-24-06867-s001.zip › sensors-3225243-supplementary.pdf]

A series of rare earth metal-based coordination polymers: Fluorescence and sensing studies

Nian-Hao Wang, Jin-Mei Liu, Bin Tan* and Zhao-Feng Wu*

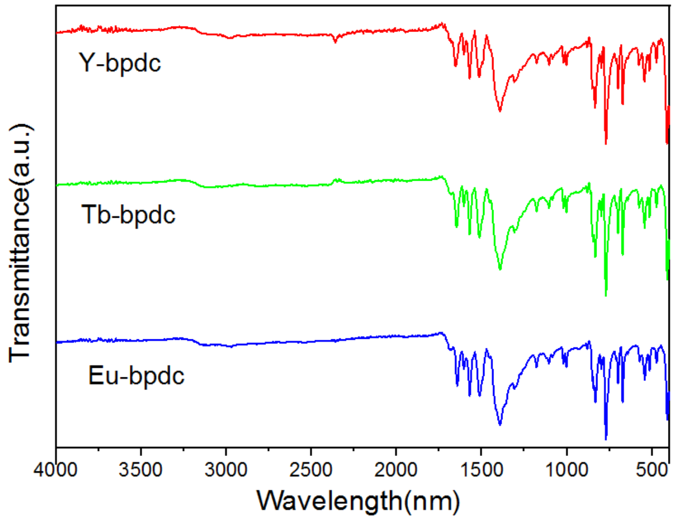

Figure S1. The FT-IR spectra for the as-made Ln-bpdc.

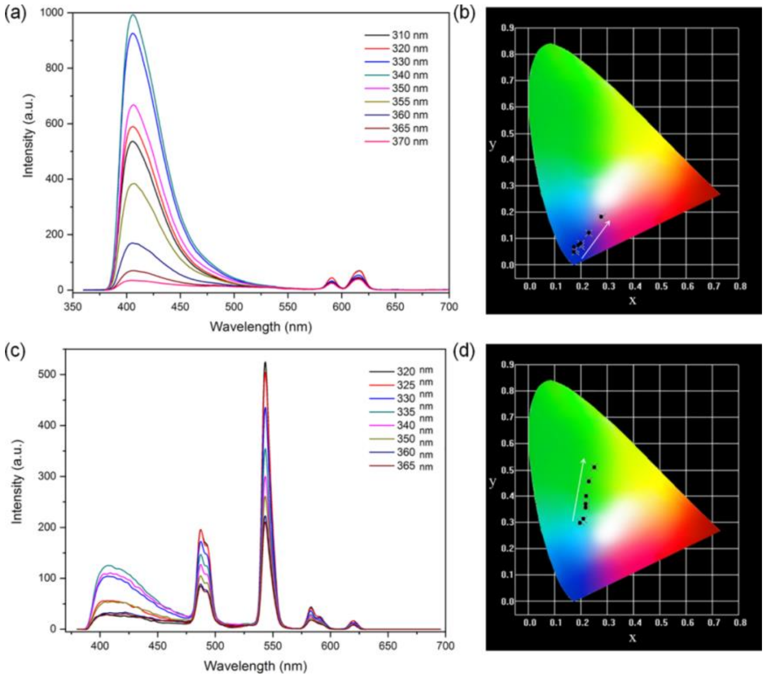

Figure S2. (a) The excitation wavelength dependent FL spectra of the as-made Eu-bpdc measured at room temperature. (b) The CIE chromaticity diagram of compound Eu-bpdc corresponding to different excitation wavelength. (c) The excitation wavelength dependent FL spectra of the as-made Tb-bpdc measured at room temperature. (b) The CIE chromaticity diagram of compound Tb-bpdc corresponding to different excitation wavelength.

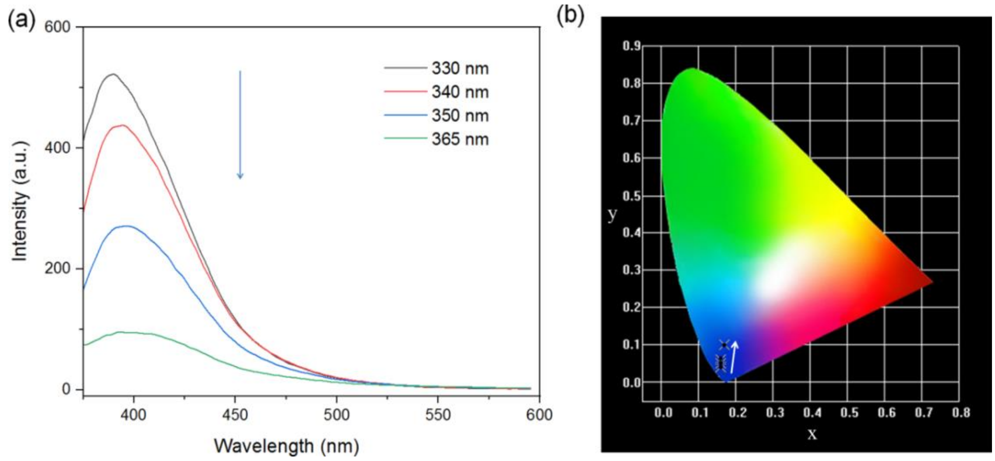

Figure S3. (a) The excitation wavelength dependent FL spectra of the as-made Y-bpdc. (b) The CIE chromaticity diagram of Y-bpdc corresponding to different excitation wavelength.

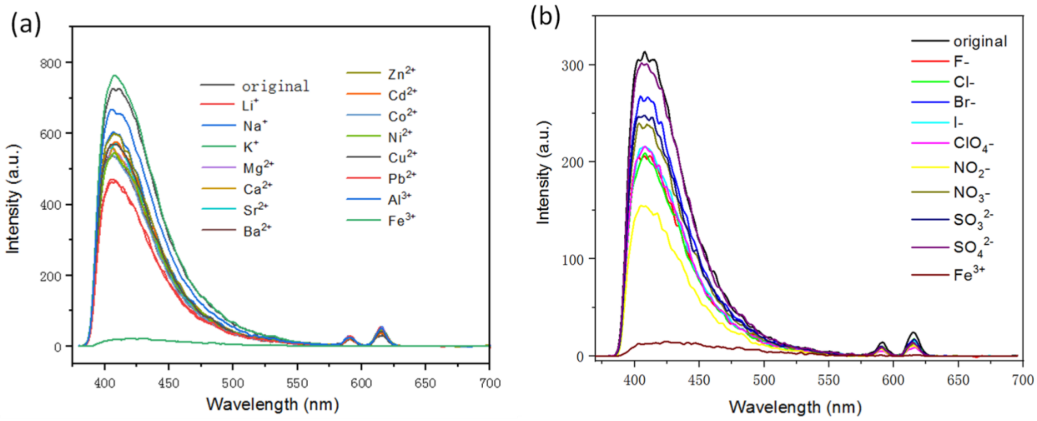

Figure S4. (a) The FL spectra of Eu-bpdc dispersed in varied metal ion solutions. (b) The FL spectra of Eu-bpdc dispersed in varied anion solutions.

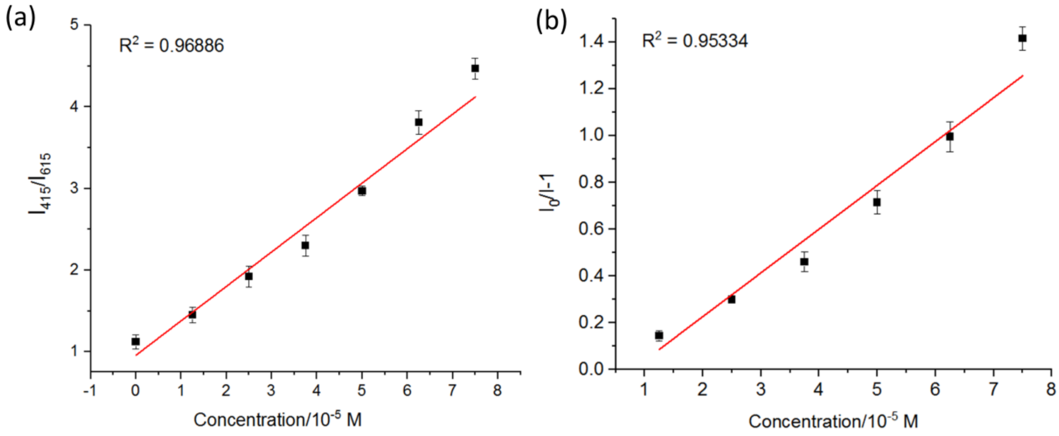

Figure S5. (a) The linear relationship between the FL intensity ratio (I_{415}/I_{615}) and the concentrations of Fe^{3+}. (b) The K_{sv} curve of Eu-bpdc towards Fe^{3+} ions by using I_{615} as a reference. The data are collected at least two times to keep accuracy.

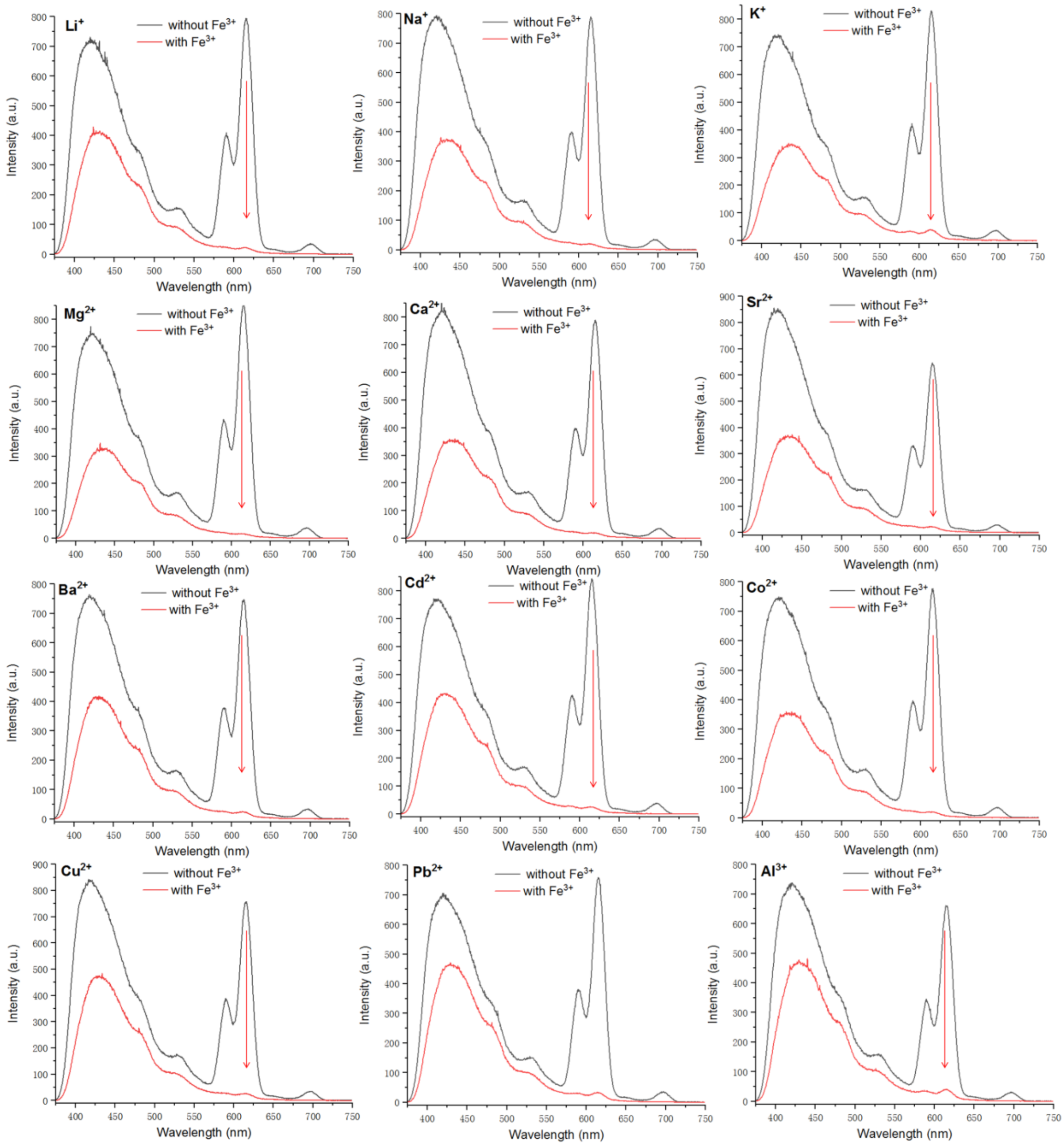

Figure S6. The FL spectra of Eu-bpdc upon addition of Fe^{3+} solution in the absence and presence of different metal cations.

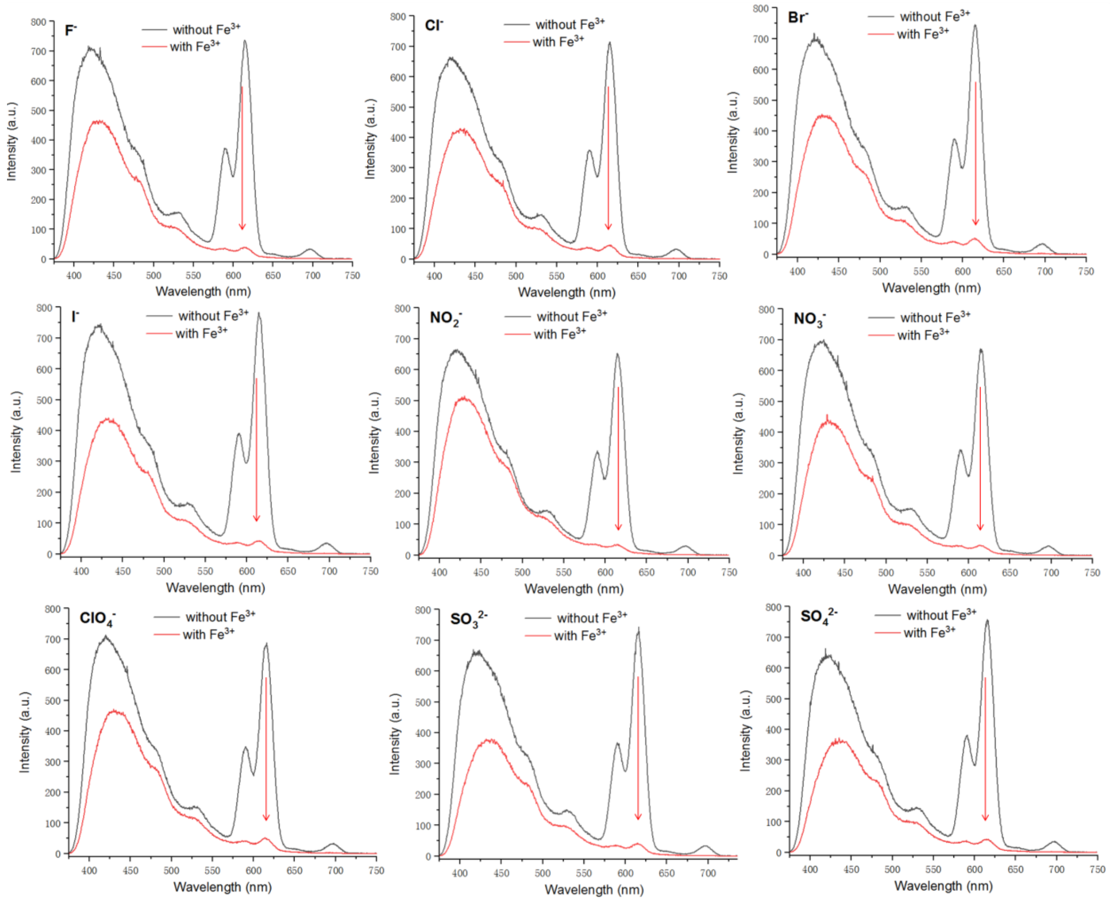

Figure S7. The FL spectra of Eu-bpdc upon addition of Fe^{3+} solution in the absence and presence of different anions.

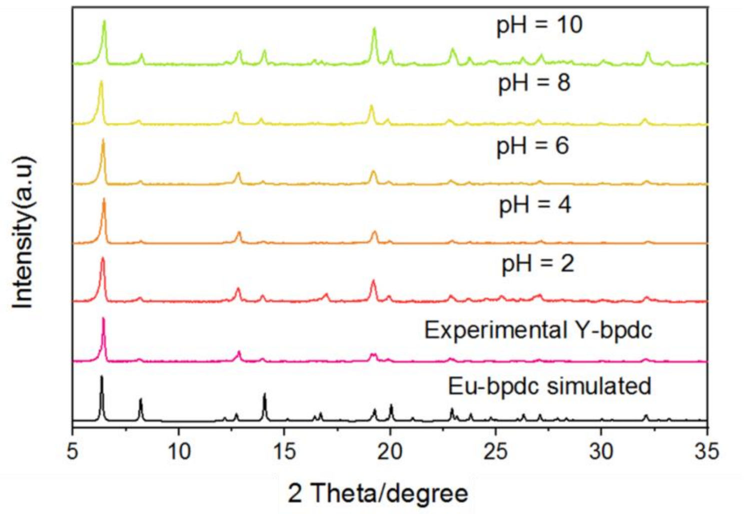

Figure S8. The PXRD of the Y-bpdc immersed in water with different pH conditions.

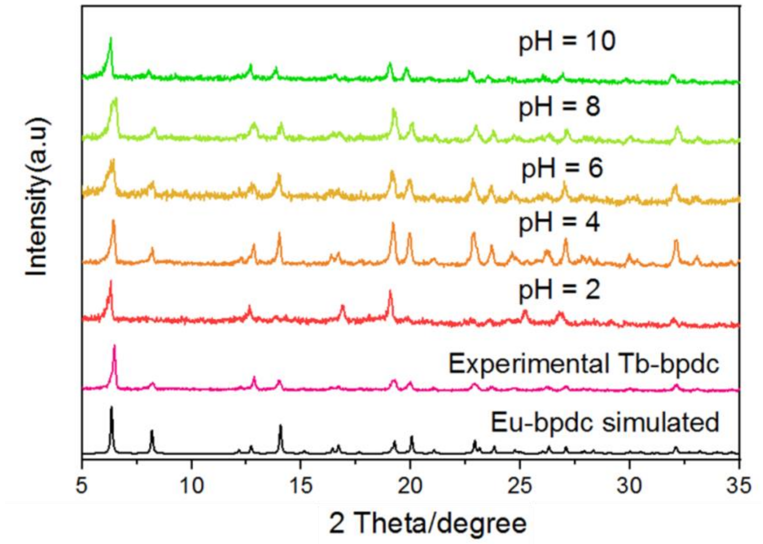

Figure S9. The PXRD of the Tb-bpdc immersed in water with different pH conditions.

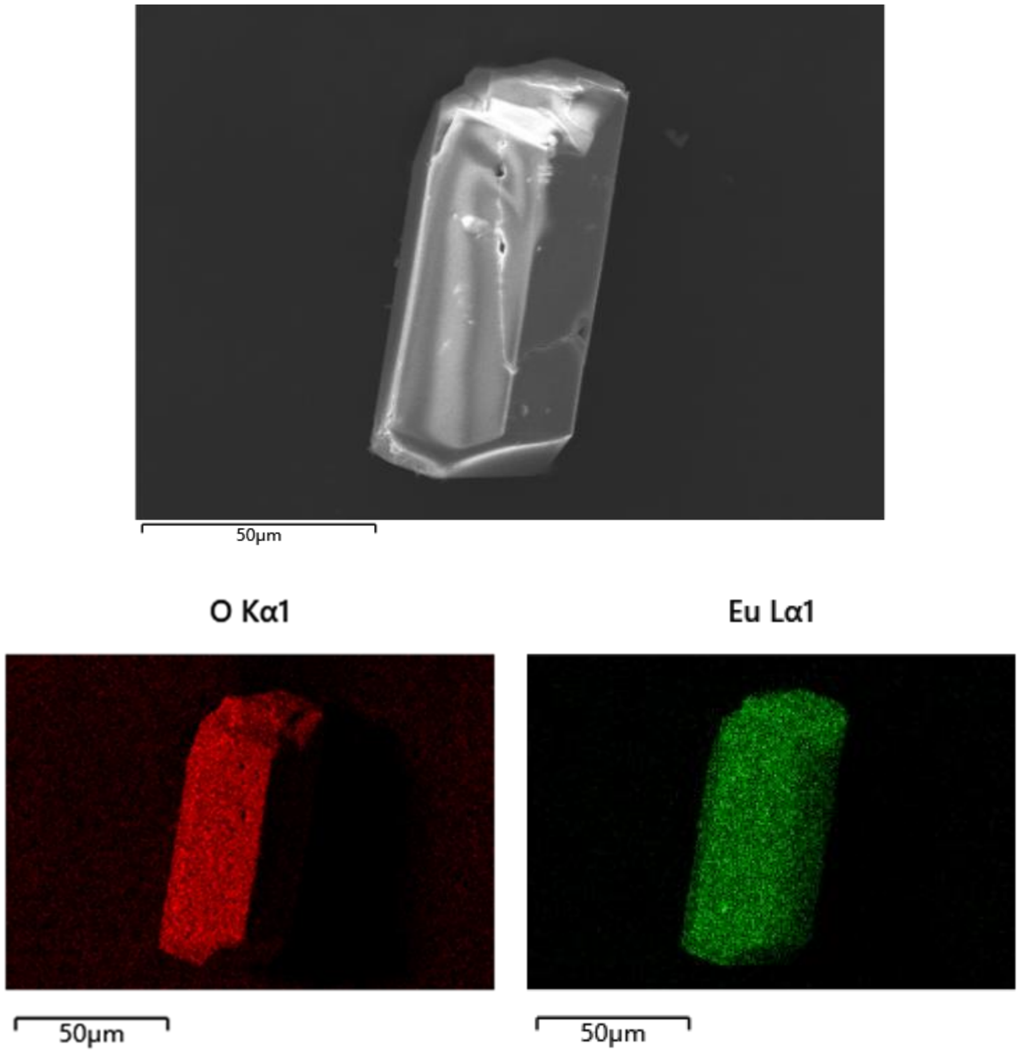

Figure S10. The EDS mapping of Eu-bpdc after Fe^{3+} sensing.
